# Safe performance of cytoreductive surgery and hyperthermic intraperitoneal chemotherapy in patients with HIV infection

**DOI:** 10.1002/cnr2.1667

**Published:** 2022-07-22

**Authors:** Anna Coghill, Julian Sanchez, Sweta Sinha, Jennifer B. Permuth, Danielle Laskowitz, Benjamin D. Powers, Sean P. Dineen

**Affiliations:** ^1^ Center for Immunization and Infection Research in Cancer Moffitt Cancer Center Tampa Florida USA; ^2^ Cancer Epidemiology Program Moffitt Cancer Center Tampa Florida USA; ^3^ Department of Oncologic Sciences, Morsani College of Medicine University of South Florida Tampa Florida USA; ^4^ Department of Gastrointestinal Oncology Moffitt Cancer Center Tampa Florida USA

**Keywords:** GI surgery, HIPEC procedure, HIV and cancer, HIV oncology

## Abstract

**Background:**

Patients with HIV (PHIV) are living longer with the adoption of anti‐retroviral therapy. As such, more patients are presenting with advanced cancer diagnoses, including peritoneal surface malignancies. The objective of this study was to assess the safety of CRS/HIPEC in this cohort of patients.

**Case:**

Five PHIV were identified, four of whom underwent CRS/HIPEC. Primary sites of disease were low‐grade appendiceal mucinous tumors in three patients and peritoneal mesothelioma in the other. Operative time ranged from 7 to 14 h. One patient developed a Clavien grade II complication postoperatively. There was no instance of neutropenia identified. One patient died of disease 19 months after surgery; the remaining three patients are alive 11, 21, and 33 months postoperatively.

**Conclusion:**

This study demonstrates that CRS/HIPEC can be performed in PHIV without prohibitive complications and operative recovery approximates that of non‐HIV patients. Though more study is needed, HIV should not preclude a patient from being offered CRS/HIPEC.

## 1 INTRODUCTION

Peritoneal metastasis can occur in patients with gastrointestinal cancers. Such metastatic spread is typically associated with a worse prognosis than metastasis to other organs, with a median overall survival of only 12 months.^1^ Systemic chemotherapy alone is also less effective than for other parenchymal‐based metastatic disease. However, the combination of systemic treatment and cytoreductive surgery with hyperthermic intraperitoneal chemotherapy (CRS/HIPEC) has demonstrated improved survival in certain subsets of patients^2^ CRS/HIPEC is an invasive surgical procedure with two components: cytoreduction involves surgical removal of all visible disease, and HIPEC involves bathing the peritoneal cavity with heated chemotherapy to treat thin or microscopic residual disease.^3^, ^4^ Candidates for CRS/HIPEC include select patients with peritoneal mesothelioma or peritoneal involvement from cancers of the appendix, colon, or stomach.^5^, ^6^, ^7^, ^8^

With the introduction of effective antiretroviral therapy (ART), patients with HIV (PHIV) are living to older ages. One consequence of this trend is that PHIV are developing age‐related, chronic comorbidities such as cancer at higher numbers.^9^, ^10^ There are no data to suggest whether PHIV are at higher risk for development of peritoneal dissemination of disease, but population‐based research consistently reports that stage‐matched cancer PHIV receive cancer treatment less frequently and experience elevated mortality compared to cancer patients without HIV.^11^, ^12^, ^13^, ^14^ Despite current National Comprehensive Cancer Network (NCCN) guidelines clearly stating that PHIV should receive equivalent cancer therapy, PHIV are often excluded from clinical trials of more novel therapeutic approaches.^15^ As CRS/HIPEC is an invasive, extensive operation, there are potential concerns about offering such a procedure to immunocompromised patients. However, general operative mortality has dramatically improved in the current HIV treatment era.^16^, ^17^ The objective of this case series is to report a contemporary experience of the safe and effective administration of CRS/HIPEC to PHIV at an NCI‐designated comprehensive cancer center.

## 2 METHODS

Moffitt Cancer Center is a large, NCI‐designated comprehensive cancer center in Southwest Florida that serves a catchment area inclusive of 2 of the 50 geographic areas classified by the Centers for Disease Control as priority jurisdictions due to contributing to more than half of new HIV diagnoses in the United States.^18^ Investigators utilized an institutional database that retrospectively identifies cancer PHIV using International Classification of Disease (ICD) codes (ICD‐9: 042–044, 079.53, 795.71, and 795.8; ICD‐10: V08, B20, B97.35, R75, O98.7, and Z21) to identify advanced stage PHIV in the gastrointestinal oncology (GI) program. The electronic medical record (EMR) was reviewed to confirm CRS/HIPEC administration, and the patient list was reviewed against the surgical log of the senior author to ensure complete patient ascertainment. Information on patient and disease features, operative details, post‐operative complications and blood counts, and HIV metrics were abstracted from the EMR. The study was performed in with an institutional IRB‐approved protocol and in concordance with Federal Policy for the Protection of Human Subjects.

The technique for CRS/HIPEC has been described, in general, previously.^4^, ^19^ Specifically, at out institution, CRS/HIPEC begins with cystoscopy and placement of bilateral ureteral catheters. The CRS phase begins with exploratory laparotomy and lysis of adhesions as necessary. Visceral resections are performed for any organs with evidence of visible disease (which include omentum, small intestine, appendix, colon, rectum, spleen, ovaries, uterus, and gallbladder). Peritoneal resections (e.g., parietal, diaphragmatic, and pelvic) are performed if gross disease is identified. Near the end of the cytoreductive phase, the operating room and patient are cooled in preparation for HIPEC. An intraperitoneal inflow and outflow catheter is placed, and the skin is closed. The catheters are connected to machine perfusion to create a closed circuit, and heated perfusion begins while the abdomen is gently agitated. Mitomycin C is the chemotherapeutic agent used (30 mg are instilled at the initiation of the procedure and an additional 10 mg after 60 min). At the completion of the HIPEC phase, the abdomen is copiously irrigated, and the skin is opened. The final phase of the operation includes the creation of anastomoses or stomas, drain placement and abdominal closure.

## 3 RESULTS

We identified five PHIV between August 2018 and May 2020 who underwent evaluation for CRS/HIPEC. One patient was found to have disease that was not amenable to complete CRS and therefore, the procedure was terminated without HIPEC. This series, therefore, included three males and one female, ranging in age from 49 to 62 years. Characteristics of the remaining four patients are outlined in Table 1. Three patients were diagnosed with low‐grade appendiceal tumors and one with peritoneal mesothelioma. The three appendiceal cancer patients had at least moderate volume of disease as evidenced by Peritoneal Cancer Index (PCI) greater than 20. Two appendiceal cancer patients had prior chemotherapy administration, and the mesothelioma patient had a prior surgical resection (i.e., CRS without HIPEC).

**TABLE 1:** Patient characteristics and operative data for patients living with HIV who underwent CRS/HIPEC (2018–2020)

Sex	Cancer type	Prior chemotherapy	Prior surgery	Pre‐op tumor markers	CRS Procedures	Operative time	Blood Products	EBL (ml)	PCI	CC Score
M	Low‐grade mucinous adenocarcinoma of the appendix	Capecitabine and oxaliplatin	No	CEA: 40.7	Greater and lesser omentectomy, splenectomy, right hemicolectomy, peritoneal resection (RUQ, LUQ, LLQ, and pelvis), splenectomy, portal dissection, right hemicolectomy, and bilateral diaphragm repair	840 min	1 U PRBCs	500	28	2
				CA 125: 34.3						
				CA 19–9: 203.2						
M	Low‐grade mucinous adenocarcinoma of the appendix	No	No	CEA: 9.9	Greater and Lesser Omentectomy, peritonectomy (RUQ, LUQ, RLQ, LLQ, and pelvis); splenectomy; and appendectomy	658 min	1 U PRBCs	600	22	1
				CA 125: wnl						
				CA 19–9: wnl						
F	Low‐grade mucinous adenocarcinoma of the appendix	FOLFOX and Avastin	No	CEA: 309.6	Greater omentectomy; Right colectomy, bilateral salpingo‐oophorectomy, and peritoneal resections (RUQ, LUQ, and pelvis)	603 min	None	600	38	2
				CA 125: 271.6						
				CA 19–9: 728.8						
M	Well‐differentiated mesothelioma	No	Yes	N/A	Greater Omentectomy, Mobilization of splenic flexure, Resection of cystic tumor; Resection of pelvic peritoneum	437 min	None	200	6	0

Abbreviations: pre‐op, 30 days pre‐operative; CEA, carcinoembryonic antigen; CA, cancer antigen 125; wnl, within normal limits; N/A, not available in the electronic medical record; CRS, cytoreductive surgery; RUQ, right upper quadrant, LUQ, left upper quadrant; LLQ, left lower quadrant; PRBC, packed red blood cells; EBL, estimated blood loss; PCI, peritoneal cancer index; and CC, Completeness of Cytoreduction Score.

**FIGURE 1:**
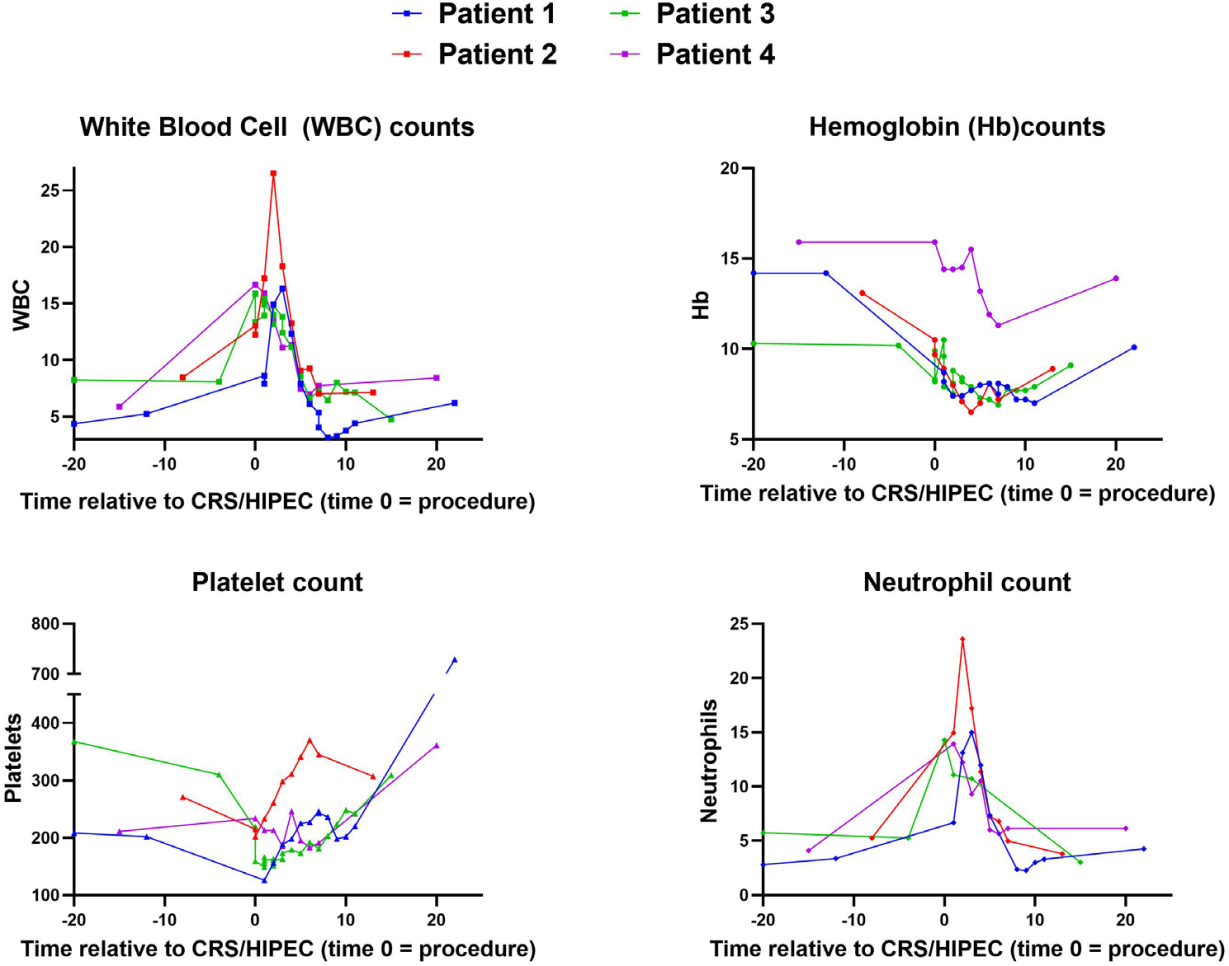
Postoperative data for white blood cell count, hemoglobin, platelets, and neutrophils for PHIV following CRS/HIPEC

The operative time ranged from 7 to 14 h. Appendiceal cancer patients with PCI scores of 28 and 38 underwent incomplete cytoreduction (Completeness of Cytoreduction Score [CC] 2 resection). However, both patients had at least moderate volume ascites that prompted HIPEC to be included to attempt to control re‐accumulation of ascites. The postoperative length of stay ranged from 8 to 12 days (Table 2). One patient developed bilateral venous thromboembolism (Clavien grade II complication).^20^ There were no 30‐day readmissions and no recorded post‐operative neutropenia, defined as absolute neutrophil count less than 1500 within 30 days of CRS/HIPEC. Complete blood cell count values recorded in the EMR prior to and in the 30‐day post‐operative window are noted in Figure 1 and demonstrate expected patterns of post‐operative variation.

**TABLE 2:** Post‐operative and HIV characteristics of patients living with HIV who underwent CRS/HIPEC (2018–2020)

Sex	Complication	Length of stay	Readmit	Post‐op Neutropenia	HIV drugs	Pre‐op HIV VL	Post‐op HIV VL	CD4+ T‐cell data	Vital Status	Follow‐up Time
M	Grade I	11 days	No	No	Genvoya	undetectable	undetectable	4mo: >12 000	Deceased	19 months
M	No	8 days	No	No	Descovy, tivicay	< 20/ml	5 mo: 44000	Pre‐op: 662	Alive with disease	33 months
							10 mo. undetectable	1mo: 800		
F	Grade II	12 days	No	No	Atripla	undetectable	N/A	1 mo: 367	Alive with disease	21 months
M	No	8 days	No	No	Biktarvy	undetectable	N/A	Pre‐op: 642	Alive, no disease	11 months
								1 mo: <200		

Abbreviations: post‐op, 30 days post‐operative; pre‐op, 30 days pre‐operative; VL, viral load; N/A, not available in the electronic medical record; and mo, month.

All patients were confirmed as being on ART prior to CRS/HIPEC. Patients experienced a transient period during which ART was not administered commensurate with the procedure, but re‐start of ART was confirmed in all patients prior to discharge. HIV RNA levels were undetectable in three patients and < 20 copies/ml in one patient prior to CRS/HIPEC. As noted in Table 2, one patient experienced a transient increase in HIV viral load 5 months post‐operatively, but this resolved to an undetectable level by 10 months. CD4+ T‐cell counts were not uniformly available in the EMR prior to CRS/HIPEC, but all patients had a CD4+ T‐cell count ≥350 cells/mm^3^ confirmed either prior to or within 6 months following the procedure. One patient with a CD4+ T‐cell count of 642 prior to therapy did drop below 200 within 1 month of CRS/HIPEC. This patient has been followed for less than 1 year and has not had a CD4+ T‐cell count update, but, importantly, the patient remains alive without any evidence of disease or HIV‐associated adverse outcomes. Three of the four patients remain alive at 11, 21, and 33 months post‐operatively. One patient with progression of low‐grade mucinous adenocarcinoma and unresectable disease transferred care to hospice and died 19 months after CRS/HIPEC, although this death was not related to HIV.

## 4 DISCUSSION

We reviewed the experience of four PHIV treated with CRS/HIPEC at Moffitt Cancer Center between August 2018 and May 2020. Patients with peritoneal mesothelioma or low‐grade mucinous adenocarcinoma of the appendix with peritoneal dissemination of disease are typically considered to be candidates for CRS/HIPEC.^21^ GI cancers are occurring at higher numbers in PHIV as effective ART has extended life expectancy for this patient group,^9^ and our report represents the largest series documenting of safe administration of CRS/HIPEC in PHIV.

Given the potential for HIPEC with Mitomycin C to lead to hematologic complications, notably leukopenia, it was unclear prior to this report whether this invasive procedure in PHIV would have severe side effects. However, we found no evidence of severe or unexpected complications in PHIV, with length of hospital stay, post‐operative lymphocyte counts, and prognosis all in line with expectations from the general oncology population. Patients developed grade I/II complications at approximately the same rate as national cohort studies,^7^ although the limited number of patients in this study prevent statistical comparisons. Two of the four patients in this cohort did undergo incomplete, CC‐2 resections; however, these patients had certain factors associated with a higher risk of incomplete cytoreduction, including male gender, ascites, and elevated preoperative tumor markers.^22^ Each of the PHIV in this case series was receiving standard ART, had well‐controlled HIV infection at the time of CRS/HIPEC, and no patient had any recorded adverse outcomes related to HIV.

One additional consideration specific to CRS/HIPEC was the potential for exposure of staff members during the perfusion portion of the procedure, during which a heated chemotherapy solution is circulated throughout the abdomen via inflow and outflow cannulas introduced into the peritoneum. During this time, staff may be affected by contact with bodily fluids. However, we recorded safe administration of the procedure and no exposures to bloodborne pathogens. In general, the risk associated with environmental exposure to HIV is low—approximately 0.3%–0.5% risk following a needle stick.^23^, ^24^ This risk can be further lowered by wearing double gloves, which is routine practice during HIPEC at our institution.^25^ We assert that exposure risks are minimal and should not preclude equivalent administration of CRS/HIPEC to PHIV.

In this case series, we did not identify risk factors or observe poor outcomes that would preclude PHIV from consideration for CRS/HIPEC. This is consistent with data in cardiac surgery and gastrointestinal surgery in which patients with well‐controlled HIV did not show significantly worse outcomes.^26^, ^27^ As PHIV continue to live longer, the number of patients with peritoneal metastasis will increase. This unique population is understudied. Previously, we identified a single case of a patient with mesothelioma treated with CRS/HIPEC.^28^ This patient was successfully treated with CRS/HIPEC. This report, though it includes only four patients, further expands the understanding that this procedure can be performed safely in the HIV population. Our data support inclusion of PHIV patients in standard treatment for this disease.

## AUTHOR CONTRIBUTIONS

**Anna Coghill:** Conceptualization (equal); data curation (equal); supervision (equal); writing—review and editing (equal). **Julian Sanchez:** Conceptualization (equal); writing—review and editing (equal). **Sweta Sinha:** Conceptualization (equal); data curation (equal). **Jennifer B. Permuth:** Conceptualization (supporting); writing—review and editing (equal). **Danielle Laskowitz:** Conceptualization (equal); writing—review and editing (equal). **Benjamin D. Powers:** Conceptualization (equal); writing—review and editing (equal). **Sean P. Dineen:** Conceptualization (equal); data curation (equal); supervision (equal); writing—original draft (equal).

## CONFLICT OF INTEREST

The authors declare no conflicts of interest.

## Data Availability

The data that support the findings of can be discussed on request from the corresponding author. The data are not publicly available due to privacy and ethical considerations.
